# Realizing High Performance in P‐Type SnBi_2_Te_4_ Through Synergistically Improving Effective Mass and Suppressing Bipolar Thermal Conductivity

**DOI:** 10.1002/advs.202506963

**Published:** 2025-07-02

**Authors:** Ke Zhao, Dongyang Wang, Tao Hong, Jiaqi Zhu, Siqi Wang, Shaobo Cheng, Xiang Gao, Chongxin Shan, Li‐Dong Zhao

**Affiliations:** ^1^ Key Laboratory of Materials Physics of Ministry of Education School of Physics Zhengzhou University Zhengzhou 450001 China; ^2^ School of Materials Science and Engineering Beihang University Beijing 100191 China; ^3^ Center for High Pressure Science and Technology Advanced Research (HPSTAR) Beijing 100094 China

**Keywords:** bipolar diffusion effect, low thermal conductivity, SnBi_2_Te_4_, thermoelectrics

## Abstract

Thermoelectric materials, which facilitate the mutual conversion between thermal and electrical energy, offer a promising alternative for sustainable energy solutions. High‐performance thermoelectric materials require excellent electrical conductivity and low thermal conductivity. Among emerging candidates, AB_2_X_4_ (A = Ge, Sn, Pb; B = Sb, Bi; X = Se, Te) compounds have garnered attention due to their unique septuple atomic layered crystal structure and poor lattice thermal conductivity. Here, the septuple atomic layered SnBi_2_Te_4_ is successfully synthesized and its thermoelectric performance significantly enhanced through isovalent elements alloying. The peak *ZT* ≈ 0.56 at 473 K and an average *ZT* ≈ 0.47 achieved over the temperature 300–673 K, which is 12 and 14 times higher than those in pristine SnBi_2_Te_4_. The incorporation of Sb and Se into *p*‐type SnBi_2_Te_4_ system significantly improves thermoelectric performance through three synergistic mechanisms: 1) enhance the electrical conductivity via effective mass enlarging, 2) suppress the bipolar thermal diffusion through bandgap widening, and 3) reduce the lattice thermal conductivity by point defect scattering. The results demonstrate that isovalent elements alloying is an effective strategy to realize the promising high performance of septuple atomic layered *p*‐type SnBi_2_Te_4_, which is applicable strategy for AB_2_X_4_ based compounds.

## Graphical abstract

Isovalent elements co‐alloying strategy achieves the promising high performance in septuple atomic layered *p*‐type SnBi₂Te₄ through enhancing effective mass, suppressing bipolar thermal diffusion, and reducing lattice thermal conductivity.

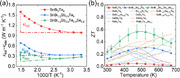

## 1 Introduction

Thermoelectric materials, capable of direct conversion between thermal and electrical energy through thermoelectric effect,^[^ ^1^, ^6^ ^]^ have emerged as a critical solution for mitigating global energy scarcity and the environmental issues stemming from excessive fossil fuel consumption. The energy conversion efficiency can be quantitatively characterized by the dimensionless figure of merit (*ZT*), which is defined as *ZT* = *S^2^σT*/*κ*_tot_. Here, *S* represents the Seebeck coefficient, *σ* denotes the electrical conductivity, *T* stands for absolute temperature in Kelvin, and *κ*_tot_ refers to the total thermal conductivity.^[^ ^7^, ^8^, ^9^ ^]^ The total thermal conductivity (*κ*_tot_) of three main components: the phonon related lattice thermal conductivity (*κ*_lat_), and the carrier contributed electronic thermal conductivity (*κ*_ele_). And particularly, the bipolar diffusion thermal conductivity (*κ*_bi_), originated from the bipolar carrier diffusion, becomes significant in narrow bandgap semiconductors. Achieving high energy conversion efficiency in thermoelectric devices demands both a high *ZT* and a high average *ZT* (*ZT*_ave_) from the thermoelectric materials. However, strong coupling relationship between *S*, *σ*, and *κ*_ele_ poses a limitation in achieving high *ZT* values. Thus, a fundamental challenge in thermoelectric research lies in decoupling the intertwined electronic transport parameters.

The advancement of thermoelectric material efficiency hinges on two critical factors: enhancing charge‐carrier mobility and suppressing lattice thermal conductivity, which are jointly addressed by the phonon‐glass electron‐crystal paradigm. The enhancement of electrical transport properties, through electronic band structure engineering,^[^ ^10^, ^11^, ^12^, ^13^, ^14^ ^]^ introducing resonant state,^[^ ^15^, ^16^, ^17^ ^]^ multi‐valley synglisis,^[^ ^18^, ^19^ ^]^ modulation doping,^[^ ^20^, ^21^, ^22^ ^]^ and lattice planification,^[^ ^23^, ^24^, ^25^ ^]^ suffers from intrinsic trade‐offs: elevated carrier densities enhance *σ* but simultaneously suppress *S* while exacerbating *κ*_ele_. Multi‐scale defect engineering through grain boundaries,^[^ ^26^, ^27^, ^28^ ^]^ nanoprecipitates,^[^ ^29^, ^30^, ^31^, ^32^ ^]^ dislocations,^[^ ^33^, ^34^ ^]^ entropy engineering,^[^ ^35^, ^36^, ^37^, ^38^ ^]^ or point defects^[^ ^39^, ^40^, ^41^, ^42^, ^43^ ^]^ has emerged as an effective strategy to reducing *κ*_lat_, though often at the expense of carrier mobility degradation. While *κ*_lat_ represents a relatively independent parameter, making its minimization critical for *ZT* enhancement.^[^ ^44^, ^45^, ^46^, ^47^, ^48^ ^]^ Consequently, the systematic exploration and design of materials with inherently low thermal conductivity represents an effective strategy to achieve superior performance.

Layered chalcogenides have garnered considerable attention since their unique crystal structure and electronic band structure, that is SnSe,^[^ ^49^, ^50^, ^51^ ^]^ SnS,^[^ ^19^, ^52^ ^]^ Bi_2_Te_3,_^[^ ^53^, ^54^, ^55^ ^]^ PbSnS_2,_^[^ ^56^ ^]^ BiCuSeO^[^ ^57^, ^58^ ^]^ and Bi_6_Cu_2_Se_4_O_6._^[^ ^59^ ^]^ The septuple atomic‐layered system AB_2_X_4_ (A = Ge, Sn, Pb; B = Sb, Bi; X = Se, Te) demonstrates an intrinsically low lattice thermal conductivity and adjustable electronic structures. These characteristics render them highly promising as potential candidates for utilization in thermoelectric applications. In SnSb_2_Te_4_, the intrinsically low thermal conductivity, which stems from pronounced lattice anharmonicity, along with the optimized carrier concentration and the increased density‐of‐state effective mass achieved through Se alloying, jointly contribute to attaining a peak *ZT* value ≈ 0.5 at 720 K.^[^ ^60^ ^]^ Qian et al. found the suppressing of bipolar diffusion effect and enhancement of electrical properties through Se alloying in PbBi_2_Te_4._^[^ ^61^ ^]^ Zhu et al. elucidate that Sb alloying effectively optimizes the electrical transport via the carrier concentration modulation and reduces the lattice thermal conductivity through strengthening point defect scattering, and achieved a peak *ZT* ≈ 0.17 at 723 K.^[^ ^62^ ^]^ The single crystal of GeSb_2_Te_4_ was obtained and the out‐of‐plane performance was optimized through Se alloying.^[^ ^63^ ^]^

SnBi_2_Te_4_, a 3D topological insulator,^[^ ^64^ ^]^ there are few reports on its thermoelectric performance since the narrow bandgap and strong bipolar diffusion effect. Pan et al. found a high concentration of *p*‐type carriers and lower carrier mobility in SnBi_2_Te_4_ results in lower Seebeck coefficient and *ZT* ≈ 0.18 at room temperature,^[^ ^65^ ^]^ which is one order higher than Zhang's result (≈ 0.013 @ 300 K)^[^ ^66^ ^]^ and lower than that *ZT* ≈ 0.3 at 400 K reported by Terzi et al.^[^ ^67^ ^]^ However, the theoretical results evidenced that a single layer of SnBi_2_Te_4_ is a promising 2D thermoelectric material, with its peak *ZT* ranging from 4.5 to 4.9 within the temperature range of 300–450 K.^[^ ^68^ ^]^ The thermoelectric performance reported in the literature varies significantly. Therefore, it is critically important to systematically evaluate and optimize the thermoelectric performance of SnBi_2_Te_4_.

In this work, we synthesized *p*‐type SnBi_2_Te_4_ samples via melting and hot‐pressing sintering methods. To address the strong bipolar diffusion effect and improve the relatively low thermoelectric performance, stepwise solid solutions with Sb and Se were adopted. These modifications aimed to enhance the electrical conductivity by optimizing carrier concentration, suppress the bipolar diffusion by widening the bandgap, increase the Seebeck coefficient by enlarging the effective mass, and reduce the lattice thermal conductivity by strengthening point defect scattering. As a results, we achieved a peak *ZT* of ≈0.56 at 473 K and an average *ZT* (*ZT*_ave_) of 0.47 over the temperature range of 300 – 673 K in the Sb and Se co‐alloyed system. This represents a 12‐ and 14‐fold improvement compared to pristine SnBi_2_Te_4_. These findings underscore the significant potential of co‐alloying strategies for enhancing the thermoelectric performance of septuple atomic layered AB_2_X_4_ compounds.

## 2 Results and Discussion

**Figure 1:**
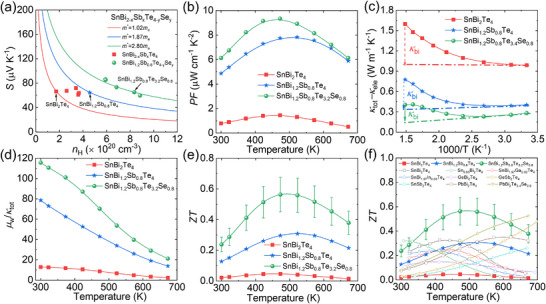
Two‐step strategy optimizes the performance of SnBi_2_Te_4_. Comparison of the transport properties in pristine, SnBi_1.2_Sb_0.8_Te_4_, and SnBi_1.2_Sb_0.8_Te_3.2_Sb_0.8_: a) Seebeck coefficient, b) power factor, c) bipolar diffusion thermal conductivity, d) the ratio of weighted mobility and total thermal conductivity, e) temperature dependent *ZT* values. The optimal *ZT* values of *p*‐type SnBi_1.2_Sb_0.8_Te_4_, and SnBi_1.2_Sb_0.8_Te_3.2_Sb_0.8_ in this work and other IV‐V_2_‐VI_4_ systems (SnBi_2_Te_4_,^[^ ^69^ ^]^ Sn_0.95_Bi_2_Te_4_,^[^ ^67^ ^]^ SnBi_1.97_Ga_0.03_Te_4_,^[^ ^69^ ^]^ SnBi_1.97_In_0.03_Te_4_,^[^ ^69^ ^]^ GeBi_2_Te_4_,^[^ ^66^ ^]^ GeSb_2_Te_4_,^[^ ^70^ ^]^ SnSb_2_Te_4_,^[^ ^71^ ^]^ PbBi_2_Te_4_,^[^ ^61^ ^]^ PbBi_2_Te_3.4_Se_0.6_^[^ ^61^ ^]^).

To address the aforementioned limitations in the performance of pristine SnBi_2_Te_4_, which are primarily attributed to three critical factors—low carrier mobility, bipolar thermal diffusion, and relatively high thermal conductivity—a two‐step alloying strategy is proposed to enhance its thermoelectric performance, as schematically displayed in **Figure** **1**. The solid solution of Sb and Se optimizes both the carrier concentration and carrier mobility, thereby significantly enhancing the electrical conductivity. The effective mass increases from ≈ 1.02*m*_e_ in pristine SnBi_2_Te_4_ to ≈ 2.80 *m*_e_ in SnBi_1.2_Sb_0.8_Te_3.2_Se_0.8_, as illustrated in Figure 1a. The maximum power factor (*PF*) is ultimately raised from ≈ 1.45 µW cm^−1^ K^−2^ in pristine SnBi_2_Te_4_ at 473 K to ≈ 9.33 µW cm^−1^ K^−2^ in SnBi_1.2_Sb_0.8_Te_3.2_Se_0.8_ (Figure 1b). The increase in carrier concentration and the broadening of the bandgap, both induced by the solid solution, significantly suppress the bipolar diffusion thermal conductivity (Figure 1c). Meanwhile, the atomic mass fluctuation and stress fluctuation caused by the solid solution enhanced phonon scattering, leading to a significant decline in the lattice thermal conductivity. The ratio between weighted carrier mobility and total thermal conductivity (*μ*_w_/*κ*_tot_) reveals that the solid solution of Sb and Se increases the *μ*_w_/*κ*_tot_ value, as displayed in Figure 1d. This indicates a synergistic optimization effect on the thermoelectric properties. Ultimately, by integrating enhanced electrical conductivity and reduced thermal conductivity, the *ZT* value across the entire temperature range is significantly improved, as shown in Figure 1e. The peak *ZT* value increases from ≈ 0.05 in pristine SnBi_2_Te_4_ to ≈ 0.56 in SnBi_1.2_Sb_0.8_Te_3.2_Se_0.8_, with the corresponding average *ZT* (*ZT*_ave_) rising from ≈ 0.03 to ≈ 0.47 over the temperature range from 300 to 673 K. Clearly higher than the reported results for AB_2_X_4_ systems, including SnBi_2_Te_4_,^[^ ^69^ ^]^ Sn_0.95_Bi_2_Te_4,_^[^ ^67^ ^]^ SnBi_1.97_Ga_0.03_Te_4_,^[^ ^69^ ^]^ SnBi_1.97_In_0.03_Te_4_,^[^ ^69^ ^]^ GeBi_2_Te_4,_^[^ ^66^ ^]^ GeSb_2_Te_4,_^[^ ^70^ ^]^ SnSb_2_Te_4,_^[^ ^71^ ^]^ PbBi_2_Te_4,_^[^ ^61^ ^]^ PbBi_2_Te_3.4_Se_0.6,_^[^ ^61^ ^]^ as illustrated in Figure 1f. This synergistic optimization strategy demonstrates the effectiveness of co‐alloying in tailoring the thermoelectric properties of SnBi_2_Te_4_, thereby providing a promising avenue for the advancement of high‐performance thermoelectric materials.

### 2.1 The Crystal Structure of Septuple Atomic Layer SnBi_2_Te_4_

The structural framework of SnBi_2_Te_4_ is derived from Bi_2_Te_3_ through the insertion of an additional Sn‐Te layer within the pentatomic layer (Te‐Bi‐Te‐Bi‐Te). Three septuple atomic layers (Te‐Bi‐Te‐Sn‐Te‐Bi‐Te) displaced along in‐plane direction and stacked along *c*‐axis of the unit cell, forming the structure of SnBi_2_Te_4_, as illustrated in Figure S1a (Supporting Information). The adjacent layers are connected via van der Waals interactions. The scanning electron microscope (SEM) image of SnBi_2_Te_4_ clearly reveals its layered structure, as shown in Figure S2 (Supporting Information). The X‐ray diffraction (XRD) pattern of SnBi_2_Te_4_ is displayed in Figure S1b (Supporting Information). All the measured diffraction peaks can be well indexed to the space group $R\bar{3}m$. The refined lattice parameters for SnBi_2_Te_4_ are *a* = *b* = 4.399 Å and *c* = 41.209 Å, as shown in Figure S1c (Supporting Information), coincidence with the reported results (*a* = *b* = 4.395 Å and *c* = 41.606 Å).^[^ ^65^, ^69^ ^]^

**Figure 2:**
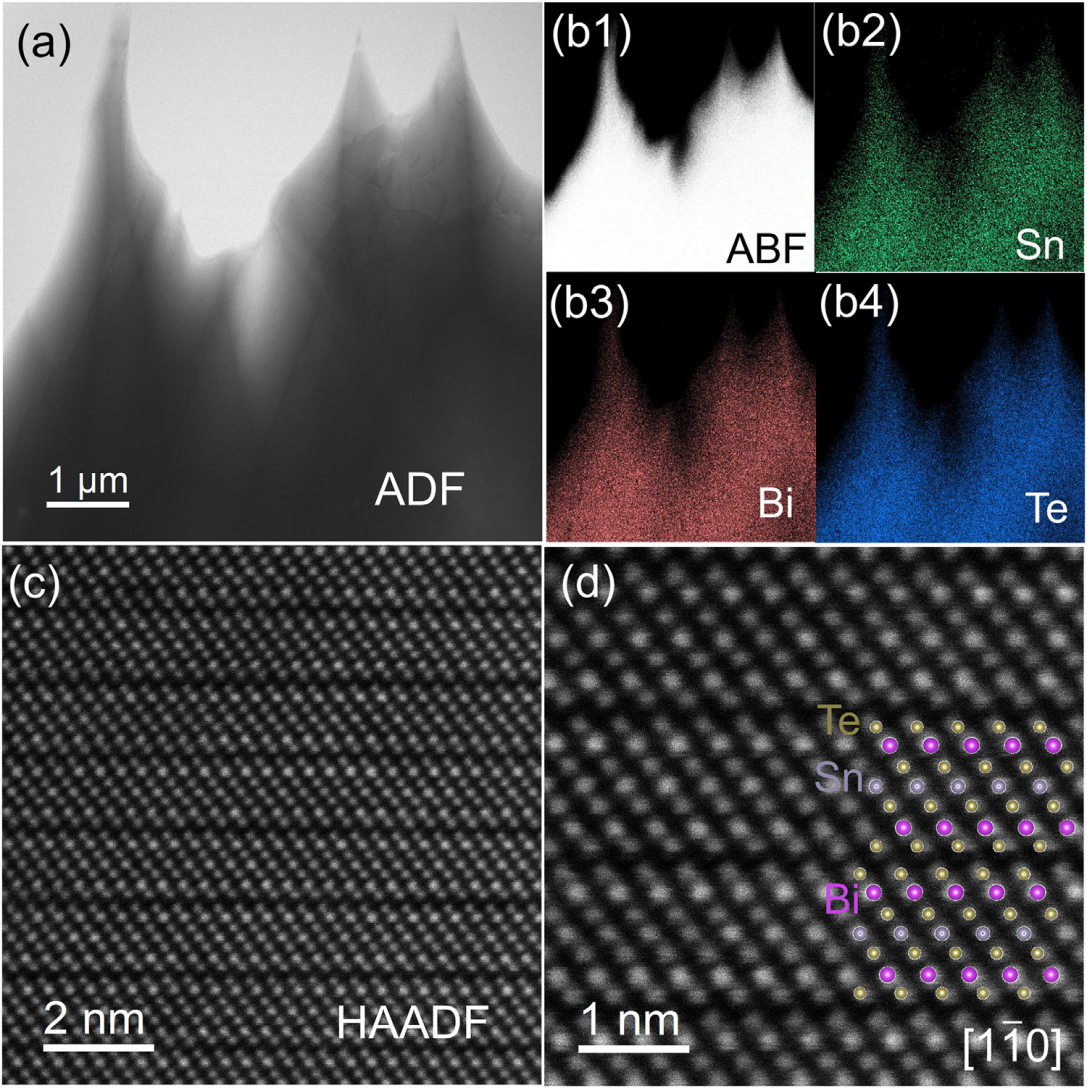
Microstructure of SnBi_2_Te_4_. a) Annular bright field (ABF) image; b1) Annular dark field (ADF) image, b2‐b4) Uniform distribution of constituent elements of the samples; c) High‐angle annular dark field (HAADF) image along [1$\bar{1}$0] direction; d) enlarged HAADF image of (c). Insert refers to the crystal structure of SnBi_2_Te_4_ along [1$\bar{1}$0] direction.

To further probe the microstructure of SnBi_2_Te_4_, Cs‐corrected scanning transmission electron microscopy (STEM) was employed, as shown in **Figure** **2**. The morphological distribution of the sample was examined using annular bright‐field (ABF) imaging (Figure 2a) and annular dark‐field (ADF) imaging (Figure 2(b1)). Energy‐dispersive X‐ray spectroscopy (EDS) analysis confirmed the homogeneous distribution of the constituent elements (Sn, Bi, and Te) within the sample (Figure 2(b2‐b4)), which indicates the high purity of the synthesized SnBi_2_Te_4_. Figure 2c shows the high‐angle annular dark field (HAADF) images acquired along [1$\bar{1}$0] direction, revealing the septuple atomic layered structure stacked along [001] direction. Due to the difference in atomic numbers (Z_Sn_ = 50, Z_Bi_ = 83, Z_Te_ = 52), a clear and strong contrast between Bi and Te atoms is observed. A magnified view of the atomic columns in Figure 2d reveals the septuple atomic layered structure (Te‐Bi‐Te‐Te‐Sn‐Te‐Bi‐Te), which is coincidence with crystal structure model projected along [1$\bar{1}$0] direction (insert in Figure 2d). These observations collectively confirm the successful synthesis of the septuple atomic layered SnBi_2_Te_4_ and provide detailed insights into its atomic‐scale structure.

### 2.2 Intrinsic Thermoelectric Transport Properties of SnBi_2_Te_4_

**Figure 3:**
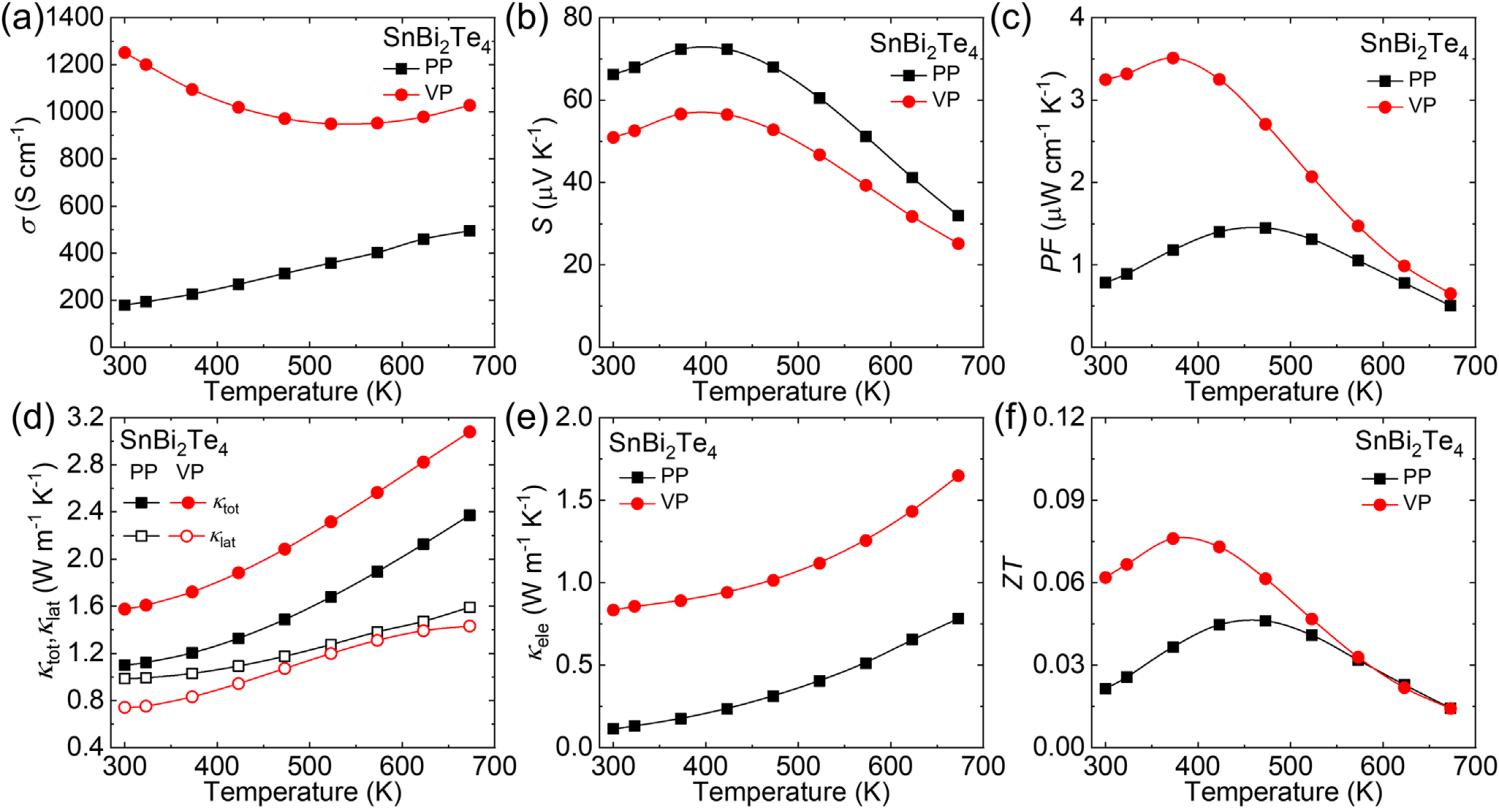
Intrinsic transport properties of SnBi_2_Te_4_ along the direction parallel (PP) and perpendicular (VP) to the pressure: a) electrical conductivity (*σ*), b) Seebeck coefficient (*S*), c) power factor (*PF*), d) total and lattice thermal conductivity (*κ*_tot_, *κ*_lat_), e) electronic thermal conductivity (*κ*_ele_), and f) figure‐of‐merit (*ZT*).

Considering the advantages of layered materials that possess low thermal conductivity, good electrical conductivity, and high thermoelectric performance, the thermoelectric performance of septuple atomic layered SnBi_2_Te_4_ was evaluated. Owing to the inherent anisotropy of the layered crystal structure,^[^ ^72^ ^]^ the thermoelectric properties of SnBi_2_Te_4_ were systematically investigated along directions parallel (PP) and perpendicular (VP) to the hot‐pressing pressure direction, as illustrated in **Figure** **3**. The room temperature electrical conductivity along parallel direction is obviously lower than that along perpendicular direction (Figure 3a), which originates from the lower carrier mobility since strong interlayer scattering.^[^ ^59^ ^]^ The positive Seebeck coefficient along the two directions suggesting the *p*‐type semiconductor character in SnBi_2_Te_4_ (Figure 3b). The Seebeck coefficient first increases and then decreases with increasing temperature, indicating the onset of the bipolar effect at the critical temperature of ≈400 K. This leads to an increase in carrier concentration due to the thermal excitation of both electrons and holes. The evaluated bandgap (*E*_g_) in SnBi_2_Te_4_ is ≈ 54 meV, which is comparable to that ≈ 50 meV measured from angle‐resolved photoemission spectroscopy (ARPES) method and ≈20 ‐ 65 meV calculated through theoretical calculations.^[^ ^64^, ^73^ ^]^ A higher carrier concentration (≈ 1.72×10^20^ cm^−3^) results in relatively lower Seebeck coefficient at room temperature. The anisotropic electrical conductivity and Seebeck coefficient give rise to the anisotropic power factor (*PF = S^2^σ*), as displayed in Figure 3c. A clear trend of initial increase followed by decrease can be observed, with the maximum *PF* reaching ≈ 1.45 W cm^−1^ K^−2^ at 473 K along the parallel direction and ≈ 3.51W cm^−1^ K^−2^ at 373 K along perpendicular direction.

The anisotropic thermal transport properties are also observed along the two directions, as displayed Figure 3d. Total thermal conductivity (*κ*_tot_) increases with increasing temperature, which is attributed to the bipolar diffusion effect and the smaller bandgap. The contribution of phonons and bipolar carriers to *κ*_tot_ can be evaluated by *κ*_lat_ + *κ*_bi_ = *κ*_tot_ ‐ *κ*_ele_. The electronic component is evaluated according to Wiedemann‐Franz relation, *κ*_ele_ = *LσT*, where *L* is Lorentz number derived from the measured Seebeck coefficient and single parabolic band (SPB) model^[^ ^50^, ^51^ ^]^ under acoustic phonon dominated scattering. At 300 K, the *κ*_tot_ along parallel direction (≈ 1.10 W m^−1^ K^−1^) is notably lower than that of the perpendicular direction (≈ 1.58 W m^−1^ K^−1^), owing to the strong scattering from interlayers, which is also found in layered compounds. The *κ*_ele_ along parallel direction is lower than that along perpendicular direction (Figure 3e), which coincides with trend for electrical conductivity. The significant disparity in electrical and thermal transport properties results in an anisotropic figure‐of‐merit (*ZT*), as illustrated in Figure 3f. A similar trend is observed between *PF* and *ZT* in both directions. A maximum *ZT* ≈ 0.08 at 473 K is obtained along perpendicular direction, which relatively smaller compared to that of SnBi_2_Se_4_ (*ZT* ≈ 0.14 @ 673 K),^[^ ^62^ ^]^ SnSb_2_Te_4_ (*ZT* ≈ 0.28 @ 720 K),^[^ ^71^ ^]^ and PbBi_2_Te_4_ (*ZT* ≈ 0.36 @ 623 K).^[^ ^61^ ^]^ Relatively lower electrical conductivity and higher thermal conductivity constrained the performance of SnBi_2_Te_4_, thus the other optimize strategies are needed to further enhance the thermoelectric performance.

### 2.3 Enhancing Electrical Properties of SnBi_2_Te_4_ Through Sb Alloying

**Figure 4:**
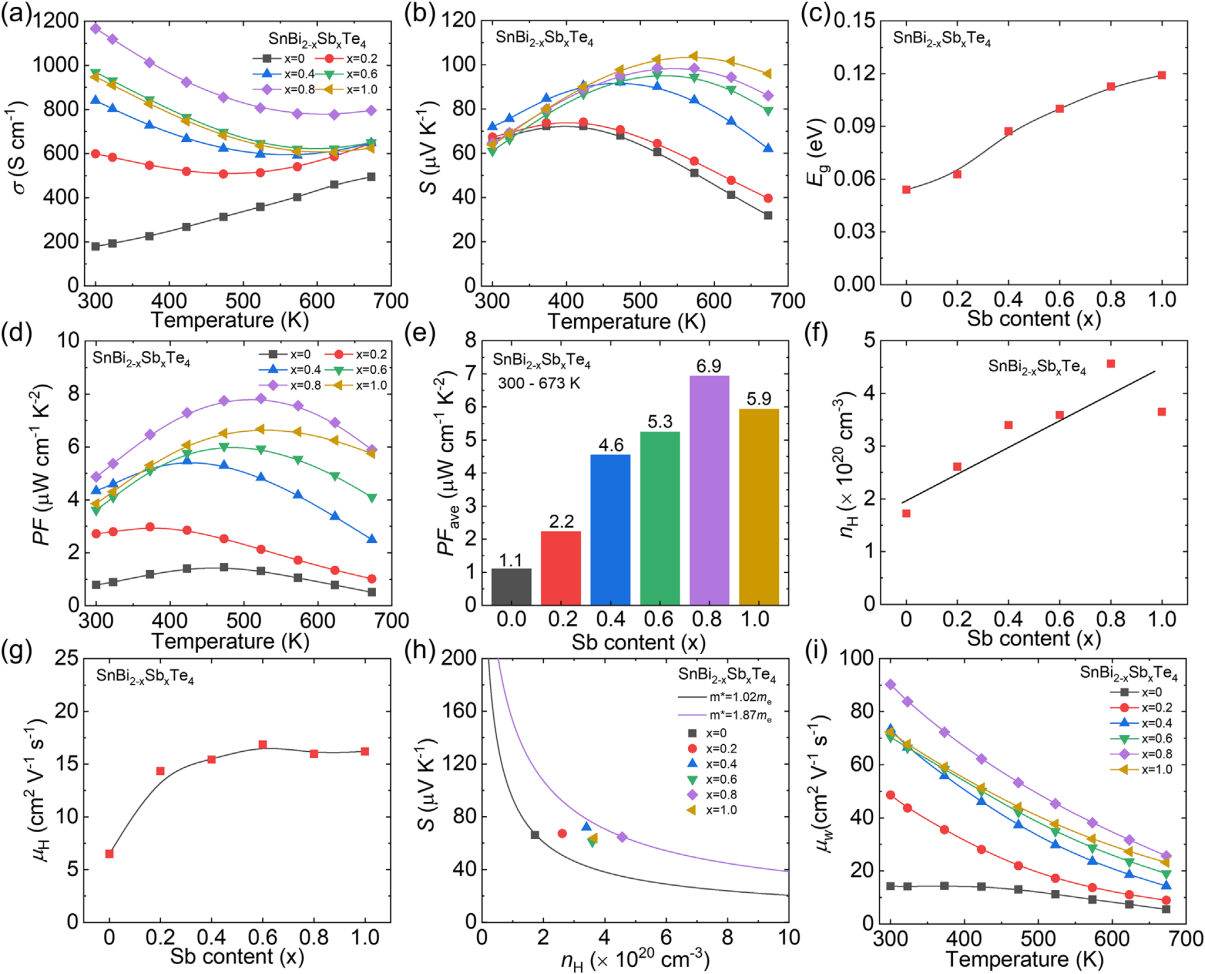
Electrical transport properties for SnBi_2‐x_Sb_x_Te_4_ (x = 0‐1.0): a) electrical conductivity (*σ*), b) Seebeck coefficient (*S*), c) bandgap (*E*_g_) calculated through *E*_g_ = 2*S*_max_ *T*_max_, d) power factor (*PF*), e) average *PF* (*PF*_ave_) within the temperature range of 300 – 673 K, f) carrier concentration and g) mobility at 300 K, h) Pisarenko relation based on SPB model, i) weighted mobility (*μ*_w_).

The anisotropic electrical transport properties of Sb alloyed SnBi_2_Te_4_ can also be found along the two directions, as displayed **Figures** **4** and S3 (Supporting Information), respectively. Electrical conductivity demonstrates a temperature‐dependent increase, indicative semiconductor behavior for intrinsic SnBi_2_Te_4_, as shown in Figure 4a. Notably, with the increase in Sb content, the electrical conductivity at room temperature significantly increases from ≈ 179 to ≈ 1167 S cm^−1^, suggesting metallic behavior since Sb alloying. Figure 4b reveals that the Seebeck coefficient remains positive across the entire temperature range, demonstrating stable *p*‐type transport characteristics. Furthermore, Sb doping induces an upward trend in the Seebeck coefficient and pushes the temperature for peak values to a higher range. The peak value enhanced from ≈ 75 µV K^−1^ at 375 K for SnBi_2_Te_4_ to ≈ 104 µV K^−1^ at 573 K for SnBiSbTe_4_. The bandgap (*E*_g_) in a bipolar system can be evaluated using the Goldsmid‐Sharp model^[^ ^74^ ^]^: *E*_g_ = 2*S*_max_ *T*_max_, where *e* is elementary charge, *S*_max_ represents the peak value of Seebeck coefficient, and *T*_max_ refers to the corresponding temperature, respectively. The increasing Sb content results in an increase in the evaluated bandgaps from ≈ 54 meV for SnBi_2_Te_4_ to ≈ 112 meV for SnBiSbTe_4_, as shown in Figure 4c, illustrating the enlargement of the bandgaps and the suppression of bipolar diffusion effect through Sb alloying. Moreover, a significant enhancement is observed at elevated temperatures, with SnBiSbTe_4_ exhibiting a threefold increase at 773 K compared to its pristine counterpart.

Integrating enhanced electrical conductivity with an improved Seebeck coefficient, a significant increase is anticipated in Sb alloyed samples. The room temperature power factor increases from ≈ 0.79 µW cm^−1^ K^−2^ for pristine SnBi_2_Te_4_ to ≈ 4.87 µW cm^−1^ K^−2^ for SnBi_1.2_Sb_0.8_Te_4_, as displayed in Figure 4d. The peak values of power factor increased from ≈ 1.45 µW cm^−1^ K^−2^ for SnBi_2_Te_4_ at 473 K to ≈ 7.83 µW cm^−1^ K^−2^ for SnBi_1.2_Sb_0.8_Te_4_ at 523 K. Additionally, the enhancement of *PF* can be found in the entire temperature range, leading to an obvious increase in average *PF* (*PF*_ave_), as illustrated in Figure 4e, which is beneficial for the larger output power density in thermoelectric devices. The average *PF* increasing from ≈ 1.11 µW cm^−1^ K^−2^ for SnBi_2_Te_4_ to ≈ 6.94 µW cm^−1^ K^−2^ for SnBi_1.2_Sb_0.8_Te_4_ over the 300–673 K temperature range, suggesting the enhancement of electrical transport properties with Sb alloying.

To understand the mechanism underlying the enhancement of electrical properties after Sb alloying, the Hall coefficients were measured. Figure 4f illustrates a slight increase in carrier concentration from ≈ 1.72 × 10^20^ cm^−3^ for the pristine to a maximum value of ≈ 4.57 × 10^20^ cm^−3^ for SnBi_1.2_Sb_0.8_Te_4_. The increasement of carrier concentration is an effective strategy to suppress the bipolar diffusion effect. Similarly, the carrier mobility (*µ*_H_) demonstrates a twofold improvement through Sb alloying and subsequently stabilizes at a plateau value with progressive Sb content incorporation, as presented in Figure 4g. Thus, the increased carrier concentration and carrier mobility results in improved electrical conductivity (Figure 4a).

Figure 4h displays the measured Seebeck coefficients of SnBi_2‐x_Sb_x_Te_4_. The Pisarenko relation was calculated based on SPB model^[^ ^50^, ^51^ ^]^ as illustrated in Figure 4h. It is found that Sb alloying leads to an increasing larger effective mass from *m* ^*^ = 1.02*m*_e_ in SnBi_2_Te_4_ to *m* ^*^ = 1.87*m*_e_ in SnBi_1.2_Sb_0.8_Te_4_. Since carrier mobility is closely related to carrier concentration and effective mass, the weighted carrier mobility (*µ*_W_) is introduced to evaluate the role of Sb in SnBi_2_Te_4_. The weighted carrier mobility,^[^ ^75^ ^]^ *µ*_W_ = *µ*_H_(*m* ^*^/*m*_e_)^3/2^, can be calculated based on the measured electrical conductivity and Seebeck coefficient, as shown in Figure 4i. The *µ*_W_ increases with Sb content and reaches its maximum in SnBi_1.2_Sb_0.8_Te_4_ over the whole temperature range, which is similar to the trend in power factor. Thus, the significant enhancement in power factor primarily stems from the synergistic optimization of carrier mobility and effective mass achieved via Sb alloying.

**Figure 5:**
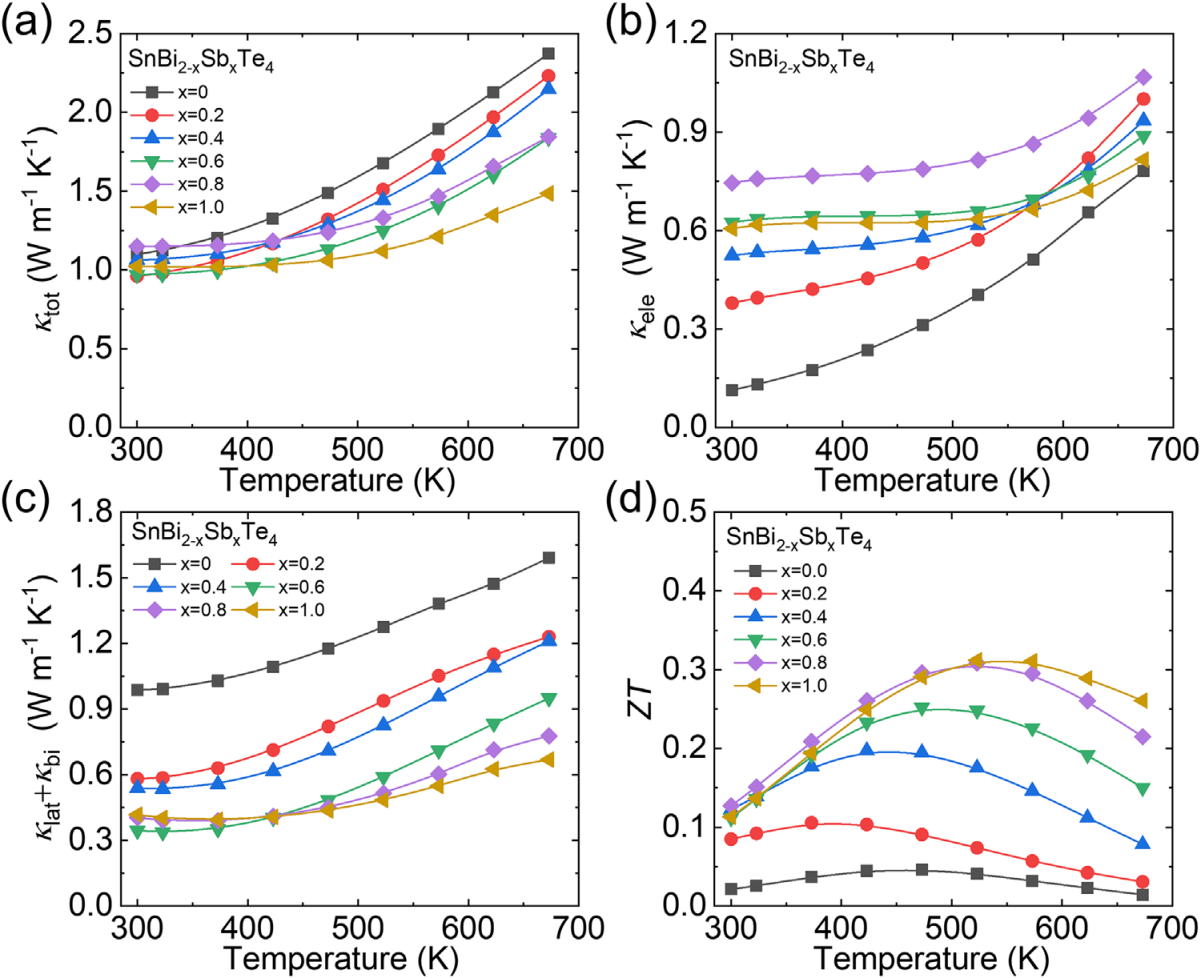
Temperature dependent thermal transport properties and performance of SnBi_2‐x_Sb_x_Te_4_ (x = 0–1.0): a) total thermal conductivity (*κ*_tot_), b) electronic thermal conductivity (*κ*_ele_), c) summation of lattice and bipolar diffusion thermal conductivity (*κ*_lat_+*κ*_bi_), and (f) figure‐of‐merit (*ZT*).

Besides, the thermal conductivity also declined through Sb alloying. As displayed in **Figure** **5**a, the total thermal conductivity (*κ*_tot_) increases with increasing temperature, indicating the strong contribution of bipolar diffusion. *κ*_tot_ decreases with increasing Sb content, especially in the higher temperature range, i.e. from ≈ 2.37 W m^−1^ K^−1^ in SnBi_2_Te_4_ to ≈ 1.48 W m^−1^ K^−1^ in SnBiSbTe_4_. The electronic thermal conductivity (*κ*_ele_) is calculated according to the Wiedemann‐Franz relation: *κ*_ele_ = *LσT*, where *L* is Lorentz number and can be obtained from measured Seebeck coefficient using the SPB model.^[^ ^50^, ^51^ ^]^ The other thermal transport properties, including heat capacity (*C*_p_), thermal diffusivity (*D*), and Lorentz number (*L*), can be found in Figure S4 (Supporting Information).

Sb alloying enhances electronic thermal conductivity, as presented in Figure 5b, which is consistent with the trend of electrical conductivity (Figure 4a). The lattice and bipolar diffusion thermal conductivities can be obtained by extracting the electronic part from total thermal conductivity: *κ*_lat_ + *κ*_bi_ =*κ*_tot_ ‐ *κ*_ele_, as shown in Figure 5c. A dramatical decrease in room temperature is mainly originated from the massive point defects scattering since the Sb alloying, i.e. from ≈ 0.98 W m^−1^ K^−1^ in SnBi_2_Te_4_ to ≈ 0.35 W m^−1^ K^−1^ in SnBi_1.4_Sb_0.6_Te_4_ at room temperature. The bipolar thermal conductivity can be effectively inhibited according to the relationship: *κ*_bi_ = *F*_bi_ *T^p^* exp(‐*E*_g_/(2*k*_B_ *T*)), where *F*_bi_ and *p* are the adjustable parameters, *E*_g_, *T*, and *k*_B_ are the bandgap, working temperature and Boltzmann constant. Qualitatively, an enlarged bandgap (Figure 4c) can effectively suppress the bipolar diffusion thermal conductivity. The decreased difference of *κ*_lat_ + *κ*_bi_ between the room and higher temperature indicating the suppressing of bipolar diffusion effect since the enhanced carrier concentration and enlarged bandgap after Sb alloying. Integrated the enhanced electrical properties and lowered thermal conductivities through Sb alloying, the figure‐of‐merit (*ZT*) was largely boosted (Figure 5d). A maximum *ZT* value ≈ 0.31 is obtained in SnBi_1.2_Sb_0.8_Te_4_ at 523 K, which is sixfold larger than that in SnBi_2_Te_4_. The corresponding average *ZT* (*ZT*_ave_) increased from ≈ 0.03 in pristine to ≈ 0.27 in SnBi_1.2_Sb_0.8_Te_4_ over 300 ‐ 673 K.

### 2.4 Thermoelectric Performance in SnBi_1.2_Sb_0.8_Te_4_ with Alloying Se

**Figure 6:**
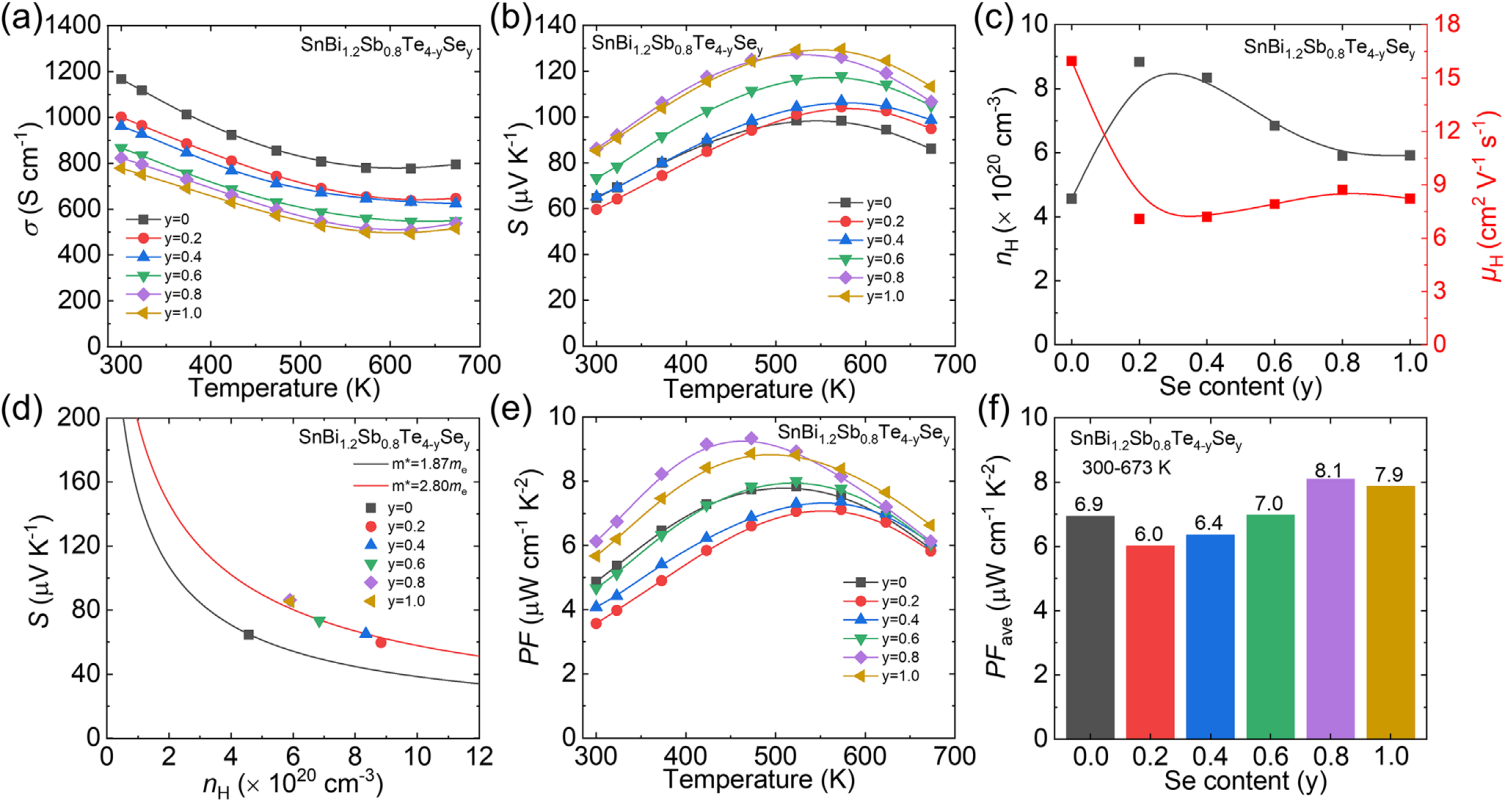
Electrical transport properties for SnBi_1.2_Sb_0.8_Te_4‐y_Se_y_ (y = 0–1.0): a) electrical conductivity (*σ*), b) Seebeck coefficient (*S*), c) carrier concentration and mobility at 300 K, d) Pisarenko relation based on SPB model, e) power factor (*PF*), f) average *PF* (*PF*_ave_) within the temperature range of 300 – 673 K.

Due to the relatively lower Seebeck coefficient of Sb alloyed samples, Se element was selected to alloying in SnBi_1.2_Sb_0.8_Te_4_ to further boost the thermoelectric performance through enhancing the electrical properties. The powder XRD patterns of SnBi_1.2_Sb_0.8_Te_4‐y_Se_y_ (y = 0 – 1.0) (Figure S5a (Supporting Information)) consistent with the PDF card, and there is no observable impurity phase can be found within the detection limit, illustrating the synthesized pure phase. The lattice parameters of the Se‐alloyed samples decrease slightly owing to the smaller atomic radius of Se compared to Te (Figure S5b, Supporting Information), which is in accordance with Vegard's Law. The transport properties were measured along the directions parallel and perpendicular to the applied pressure. The temperature‐depending thermoelectric properties along parallel direction are shown in **Figure** **6**, while the results along perpendicular direction can be found in Figure S6 (Supporting Information). The electrical conductivity decreases with increasing Se content, that is from ≈ 1167 S cm^−1^ in SnBi_1.2_Sb_0.8_Te_4_ to ≈ 778 S cm^−1^ in SnBi_1.2_Sb_0.8_Te_3_Se at 300 K, which can be attributed to the enhanced scattering since increased Se defect. The decrease first and then increase of electrical conductivity with increasing of temperature illustrates the existence of bipolar effect. Different from electrical conductivity, the Seebeck coefficient shows a significant improvement over the entire temperature range, as presented in Figure 6b. Specifically, the Seebeck coefficient increases from ≈ 64.5 µV K^−1^ for SnBi_1.2_Sb_0.8_Te_4_ to ≈ 86.2 µV K⁻¹ for SnBi_1.2_Sb_0.8_Te_3_Se at room temperature. The bandgaps, calculated based on *E*_g_ = 2*S*_max_ *T*_max_, enlarged form ≈ 0.10 eV in SnBi_1.2_Sb_0.8_Te_4_ to ≈ 0.15 eV in SnBi_1.2_Sb_0.8_Te_3_Se (Figure S7, Supporting Information), illustrating the suppression of bipolar diffusion effect.

To address the mechanism of optimization of electrical properties, the Hall coefficient was measured, as shown in Figure 6c. A clear increase trend of carrier concentration can be observed after Se alloying, while the carrier mobility decreased first and then kept as a constant with increasing Se content. Figure 6d displays the relation between Seebeck coefficient and corresponding carrier concentration at room temperature. Obviously, the Seebeck coefficients of Se alloyed samples boost from the SnBi_1.2_Sb_0.8_Te_4_, indicating the enhancement of the effective mass from ≈ 1.92*m*_e_ in SnBi_1.2_Sb_0.8_Te_4_ to ≈ 2.80*m*_e_ in Se alloyed samples based on the SPB model.^[^ ^50^, ^51^ ^]^ The increased effective mass decreases the carrier mobility (Figure 6c) and leads to a decreased electrical conductivity (Figure 6a). The decreased electrical conductivity and increased Seebeck coefficient boosting the optimized power factor, especially at the medium temperature, as presented in Figure 6e. The *PF* first increases and then decreases with increasing temperature, reaching a maximum value of ≈ 9.3 µW cm^−2^ K^−1^ for SnBi_1.2_Sb_0.8_Te_3.2_Se_0.8_ at 473 K, which is 20% higher than that in SnBi_1.2_Sb_0.8_Te_4_. The maximum *PF*_ave_ ≈ 8.1 µW cm^−2^ K^−1^ was achieved in SnBi_1.2_Sb_0.8_Te_3.2_Se_0.8_ over the temperature range of 300–673 K, as illustrated in Figure 6f.

**Figure 7:**
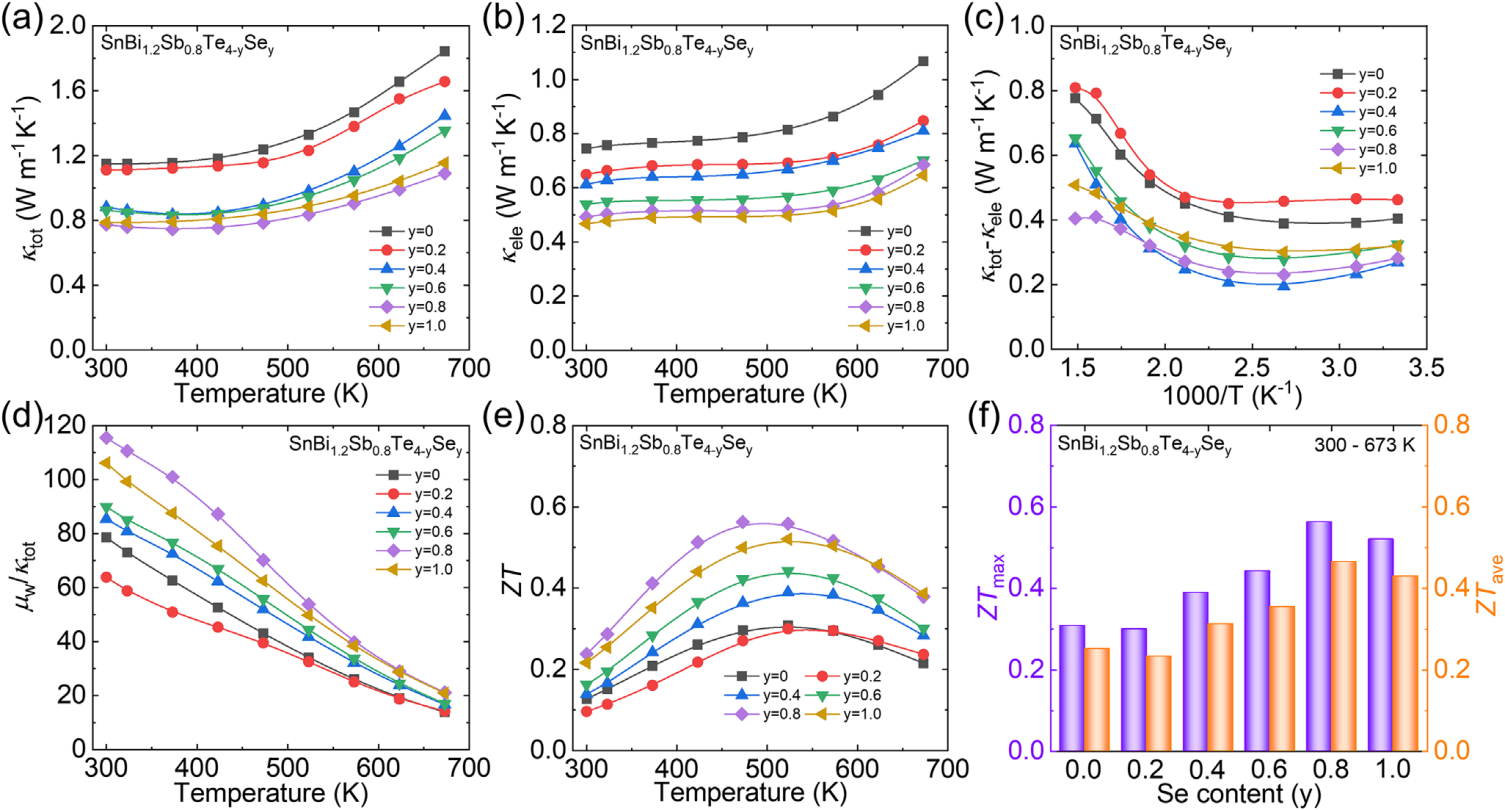
Temperature dependent thermal transport properties and performance of SnBi_1.2_Sb_0.8_Te_4‐y_Se_y_ (y = 0–1.0): a) total thermal conductivity (*κ*_tot_), b) electronic thermal conductivity (*κ*_ele_), c) summation of lattice and bipolar diffusion thermal conductivity (*κ*_lat_+*κ*_bi_) as function of 1000/T, d) the ratio (*µ*_w_/*κ*_tot_) of weighted mobility and total thermal conductivity, e) figure‐of‐merit (*ZT*), and f) peak *ZT* (*ZT*_max_) and average *ZT* (*ZT*_ave_) within the temperature range of 300 – 673 K.

The anisotropic thermal transport properties of SnBi_1.2_Sb_0.8_Te_4‐y_Se_y_ (y = 0–1.0) displayed in **Figures** **7** and S8 (Supporting Information). The total thermal conductivity, a clear decline can be observed after Se alloying and the difference between 300 and 673 K is also decreased with increasing Se content, as illustrated in Figure 7a, which is contributed by the electronic, lattice as well as bipolar diffusion parts. The electronic thermal conductivity displays a similar decreasing trend (Figure 7b) as the electrical conductivity (Figure 6a), in accordance with the Wiedemann‐Franz relation. The lattice and bipolar diffusion thermal conductivity, obtained by *κ*_lat_ + *κ*_bi_ =*κ*_tot_ ‐ *κ*_ele_, as function of 1/T is shown in Figure 7c. The lattice thermal conductivity is proportion to 1/T and presents the linear relationship, with the deviation from the line suggesting the contribution of bipolar diffusion. Obviously, the bipolar diffusion thermal conductivity decreases with increasing Se content at higher temperatures. The lattice thermal conductivity decreased from ≈ 0.41 W m^−1^ K^−1^ in SnBi_1.2_Sb_0.8_Te_4_ to ≈ 0.27 W m^−1^ K^−1^ in SnBi_1.2_Sb_0.8_Te_3.6_Se_0.4_ at 300 K, which originated from the mass and stress fluctuations induced by Se alloying.

Considering the simultaneous enhancement of the electrical properties and suppression of the total thermal conductivity, including electronic, lattice and bipolar diffusion, *µ*_w_/*κ*_tot_ was calculated and displayed in Figure 7d. The *µ*_w_/*κ*_tot_ increase with increasing Se content and achieve its peak values in SnBi_1.2_Sb_0.8_Te_3.2_Se_0.8_, illustrating the synergistic optimization of the electrical and thermal transport properties. The final *ZT* of Se alloyed substantially enhanced, as displayed in **Figure** **7**e f,. The maximum *ZT* increase from ≈ 0.31 in SnBi_1.2_Sb_0.8_Te_4_ at 523 K to ≈ 0.56 in SnBi_1.2_Sb_0.8_Te_3.2_Se_0.8_ at 473 K. Clearly higher than the reported results for AB_2_X_4_ systems, as shown in Figure 1f, including SnBi_2_Te_4,_^[^ ^69^ ^]^ Sn_0.95_Bi_2_Te_4,_^[^ ^67^ ^]^ SnBi_1.97_Ga_0.03_Te_4,_^[^ ^69^ ^]^ SnBi_1.97_In_0.03_Te_4_,^[^ ^69^ ^]^ GeBi_2_Te_4_,^[^ ^66^ ^]^ GeSb_2_Te_4_,^[^ ^70^ ^]^ SnSb_2_Te_4_,^[^ ^71^ ^]^ PbBi_2_Te_4_,^[^ ^61^ ^]^ PbBi_2_Te_3.4_Se_0.6_.^[^ ^61^ ^]^ The enhancement of *ZT* values across the entire temperature range leads to a substantial improvement in the average *ZT* (*ZT*_ave_). Specifically, SnBi_1.2_Sb_0.8_Te_3.2_Se_0.8_ (≈ 0.47) exhibits a 14‐fold increase in *ZT*_ave_ compared to pristine SnBi_2_Te_4_ (≈ 0.03) at the temperature range from 300 to 673 K. Thus, the integrated synergistic strategies, including optimizing the effective mass, suppressing the bipolar diffusion and reducing lattice thermal conductivity through solid solution of isovalent elements are effective in enhancement of the performance in septuple atomic layered SnBi_2_Te_4_.

## 3 Conclusion

In summary, we have successfully synthesized septuple atomic layered SnBi_2_Te_4_ and enhanced its thermoelectric performance through isovalent elements alloying. This work clearly demonstrates that the introduction of Sb and Se can effectively enhance the electrical properties via increasing the effective mass, suppresses the bipolar diffusion thermal conductivity through enlarging the bandgap, and reduce the lattice thermal conductivity by mass and size fluctuations in *p*‐type SnBi_2_Te_4_ system. Ultimately, we have achieved an overall improvement of the *ZT* value across the entire working temperature range, with a maximum *ZT* reaching 0.56 at 473 K and an average *ZT* of 0.47 over 300 – 673 K, which are 12 and 14 times higher than those of pristine SnBi_2_Te_4_, respectively. Our work highlights the effectiveness of synergistic strategies introduced by the solid solution of isovalent elements for improving the thermoelectric performance in septuple atomic layered SnBi_2_Te_4_, which is also applicable to AB_2_X_4_ system.

## 4 Experimental Section

The high‐purity elemental Sn (shot, 99.999%), Bi (shot, 99.999%), Te (shot, 99.99%), Sb (shot, 99.999%), and Se (shot, 99.999%) were selected to synthesis the Sb and Se alloyed SnBi_2_Te_4_ via melting and hot‐pressuring methods. The Seebeck coefficient and electrical conductivity were simultaneously measured using a ZEM‐3 instrument in a low‐pressure helium atmosphere from room temperature to 673 K. The thermal diffusivity was measured by laser flash diffusivity method using Netzsch LFA 457. More experimental details on microstructure characterization, powder XRD, Hall measurement, and the calculation method for weighted mobility and average *ZT* values can be found in Supporting Information.

## Conflict of Interest

The authors declare no conflict of interest.

## Supporting information



Supporting Information

## Data Availability

The data that support the findings of this study are available in the supplementary material of this article.
