# Supplementary material for: Realizing High Performance in P‐Type SnBi2Te4 Through Synergistically Improving Effective Mass and Suppressing Bipolar Thermal Conductivity
Source: Adv Sci (Weinh). 2025 Jul 2;12(37):e06963. doi: 10.1002/advs.202506963 (PMC12499484; doi:10.1002/advs.202506963)
Supplement: Supplementary file 1 — Supporting Information [file ADVS-12-e06963-s001.docx]

Supporting Information

Realizing High Performance in P-type SnBi_2_Te_4_ through Synergistically Improving Effective Mass and Suppressing Bipolar Thermal Conductivity

*Ke Zhao^1#^, Dongyang Wang^1#,*^,* *Tao Hong^2^, Jiaqi Zhu^1^, Siqi Wang^2^, Shaobo Cheng^1*^, Xiang Gao^3^, Chongxin Shan^1^,* *Li-Dong Zhao^2*^*

*^1^* *Key Laboratory of Materials Physics of Ministry of Education*, *School of Physics, Zhengzhou University, Zhengzhou 450001, China*

*^2^* *School of Materials Science and Engineering, Beihang University, Beijing 100191, China*

*^3^* *Center for High Pressure Science and Technology Advanced Research (HPSTAR), Beijing 100094, China*

**Experimental details**

1. **Raw materials and synthesis method**

High-purity elemental Sn (shot, 99.999%), Bi (shot, 99.999%), Te (shot, 99.99%), Sb (shot, 99.999%), and Se (shot, 99.999%) were weighed according to the stoichiometric ratios and placed into quartz tubes, which were then flame-sealed under vacuum. These tubes were subsequently placed in a muffle furnace and slowly heated to 973 K over a period of 7 hours. The samples were held at this temperature for 12 hours before being cooled at a rate of 1.7 K min^-1^ to 873 K, followed by slow cooling to room temperature. The melt-grown samples were then crushed into powders and sieved through a 160-mesh screen. The powders were subsequently sintered by hot pressing using a cylindrical graphite mold with a diameter of 12.7 mm, under the conditions of 773 K and 50 MPa for 7 minutes.

**2. Structure characterization**

The phase composition of the samples was characterized by X-ray diffraction (XRD) using Empyrean X-ray diffractometer (PANalytical, Netherlands) with Cu Kα (λ = 1.5418 Å) radiation operating at 45 kV and 40 mA.

The Scanning electron microscopy (SEM) and energy dispersive spectrometer (EDS) analysis were carried out by using a FEI Dual Beam System SEM (zeiss, Auriga) with an acceleration voltage of 20 KV.

**3. Electrical transport properties measurement**

The obtained cylindrical were cut and polished into bars with the dimensions of ~3×3×10 mm^3^ for the measurement of Seebeck coefficient and electrical conductivity by the ZEM-3 instrument (ADVANCE RIKO, Inc., Japan). The measurements were carried out in a helium atmosphere at 300 K – 773 K. The samples were coated with a thin layer of boron nitride to prevent possible evaporation. The uncertainty of the Seebeck coefficient and electrical conductivity measurement is within 5%.

**4. Hall coefficients measurements**

The Hall Coefficient *R*_H_ of the sample was measured with the instrument ET-9000. The sample used was a square sheet with a width of ~ 10 mm and a thickness of ~ 1.0 mm. The material carrier concentration *n*_H_ according to **Eq. S1**:

$n_{H}=\frac{1}{eR_{H}}$ (S1)

where *e* is amount of elementary charge. and carrier mobility (*μ*_H_) was calculated using the relationship *μ*_H_ = *σR_H._*

**5. Thermal transport properties measurements**

A shape of ~ 10 mm × 10 mm × 1 mm sample was polished and coated with a thin layer of graphite to minimize errors for the measurements of thermal diffusivity *D* using an instrument LFA 467 (NETZSCH, Germany). The total thermal conductivity was calculated by the relation *κ*_tot_ *= DC_p_ρ*, where *C_p_* is heating capacity calculated by Debye model, and *ρ* is the density of the sample measured using the Archimedes method (**Table. S1**). The lattice thermal conductivity *κ*_lat_ is subsequently determined by directly subtracting electronic thermal conductivity *κ*_ele_ from *κ*_tot_. *κ*_ele_ can be calculated by Lorenz number *L*, electrical conductivity *σ* and absolute temperature *T*, following the relation *κ*_ele_ = *LσT*. Among them, the Lorenz number *L* can be given by **Eq. S2**^[1-3]^:

$L=\left(\frac{k_{B}}{e}\right)^{2}\left\{\frac{\left(r+7/2\right)F_{r+5/2}\left(\delta\right)}{\left(r+3/2\right)F_{r+1/2}\left(\delta\right)}-\left[\frac{\left(r+5/2\right)F_{r+3/2}\left(\delta\right)}{\left(r+3/2\right)F_{r+1/2}\left(\delta\right)}\right]^{2}\right\}$ (S2)

where *k_B_* is the Boltzmann constant, *e* is the electron charge, *r* is the scattering rate, and *δ* refers to the reduced Fermi energy, which can be derived from the measured Seebeck coefficients with consideration of acoustic phonon dominated scattering (*r* = -1/2) via **Eq. S3**:

$S=\frac{k_{B}}{e}\left[\frac{\left(r+5/2\right)F_{r+3/2}\left(\delta\right)}{\left(r+3/2\right)F_{r+1/2}\left(\delta\right)}-\delta\right]$ (S3)

where *F_x_*(*δ*) is Fermi integral shown in **Eq. S4**:

$F_{x}\left(\delta\right)=\int_{0}^{\infty} \frac{\varepsilon^{x}}{1+\exp\left(\varepsilon-\delta\right)}d\varepsilon$ (S4)

Considering the parameters above, the uncertainty of the thermal conductivity is estimated to be within 8%. Considering the uncertainties from all thermoelectric parameters, the uncertainty for the final *ZT* is about 20%.

**6. Calculation of weighted mobility (*μ_W_*)**

The weighted mobility (*μ*_W_) is evaluated by *μ*_W_ = *μ*(*m**/*m*_e_)^3/2^, where me denotes the unit mass of an electron. With measured Seebeck coefficient and electrical conductivity, the *μ*_W_ could be defined as^[4]^:

$\mu_{W}=\frac{3\sigma}{8\pi eF_{0}\left(\delta\right)}\left(\frac{h^{2}}{2m_{e}k_{B}T}\right)^{3/2}$ (S5)

and combining **Eq. S3** and **Eq. S4** the *μ*_W_ can be calculated.

**7. Average *ZT* (*ZT*_ave_)**

Among a given temperature range (300–773 K), the average *ZT* value (*ZT*_ave_) is given by^[5]^:

$ZT_{ave}=\frac{1}{T_{h}-T_{c}}\int_{T_{c}}^{T_{h}} ZTdT$ (S6)

where *T*_h_ and *T*_c_ are the applied temperatures on the thermoelectric materials at high temperature and low temperature ends, respectively.


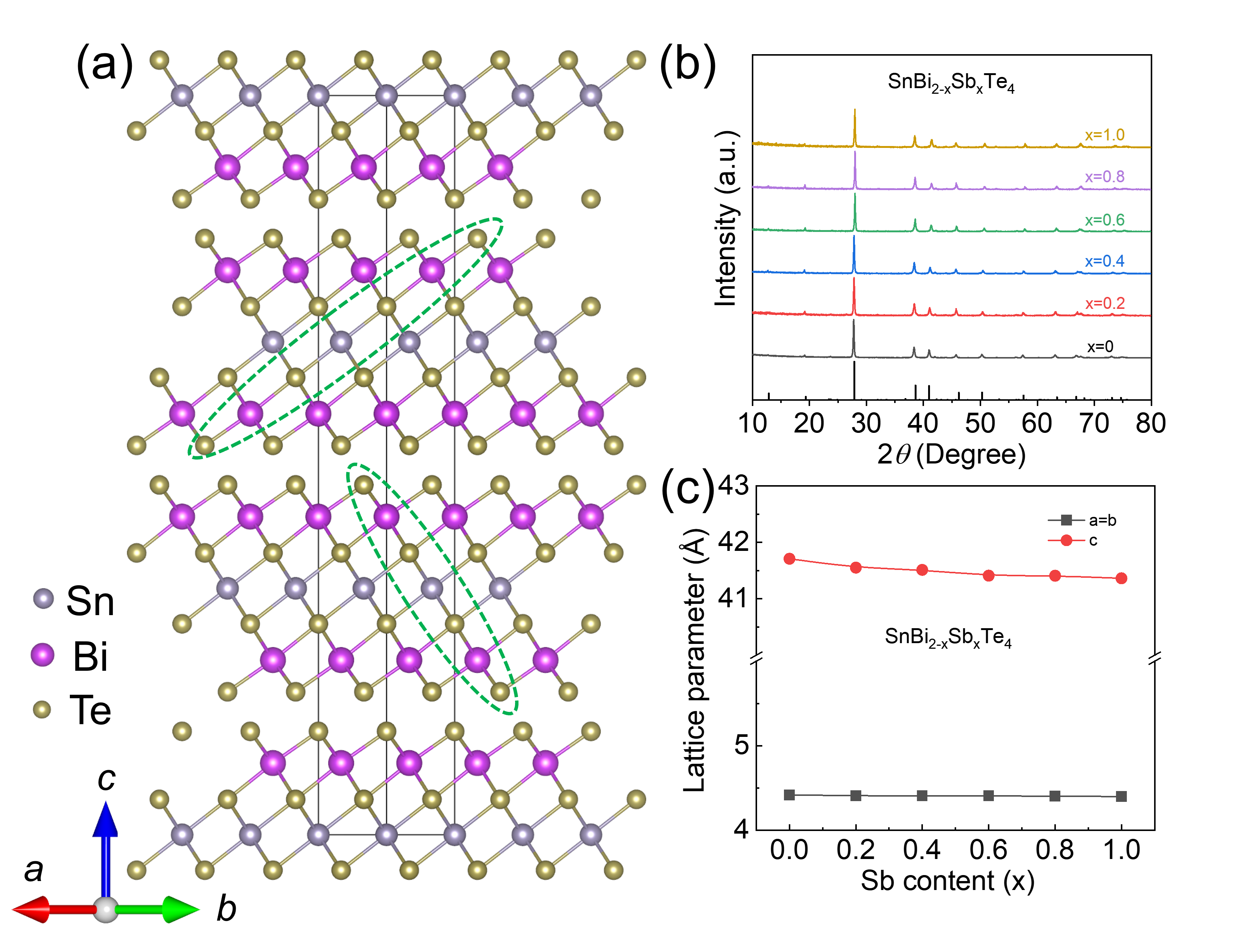


**Figure S1**. (a) The crystal structure of SnBi_2_Te_4_. The green circle refers to the septuple atomic layer of Te-Bi-Te-Sn-Te-Bi-Te. (b) Powder XRD patterns and (c) refined lattice parameters of SnBi_2-x_Sb_x_Te_4_ (x = 0 - 1.0).


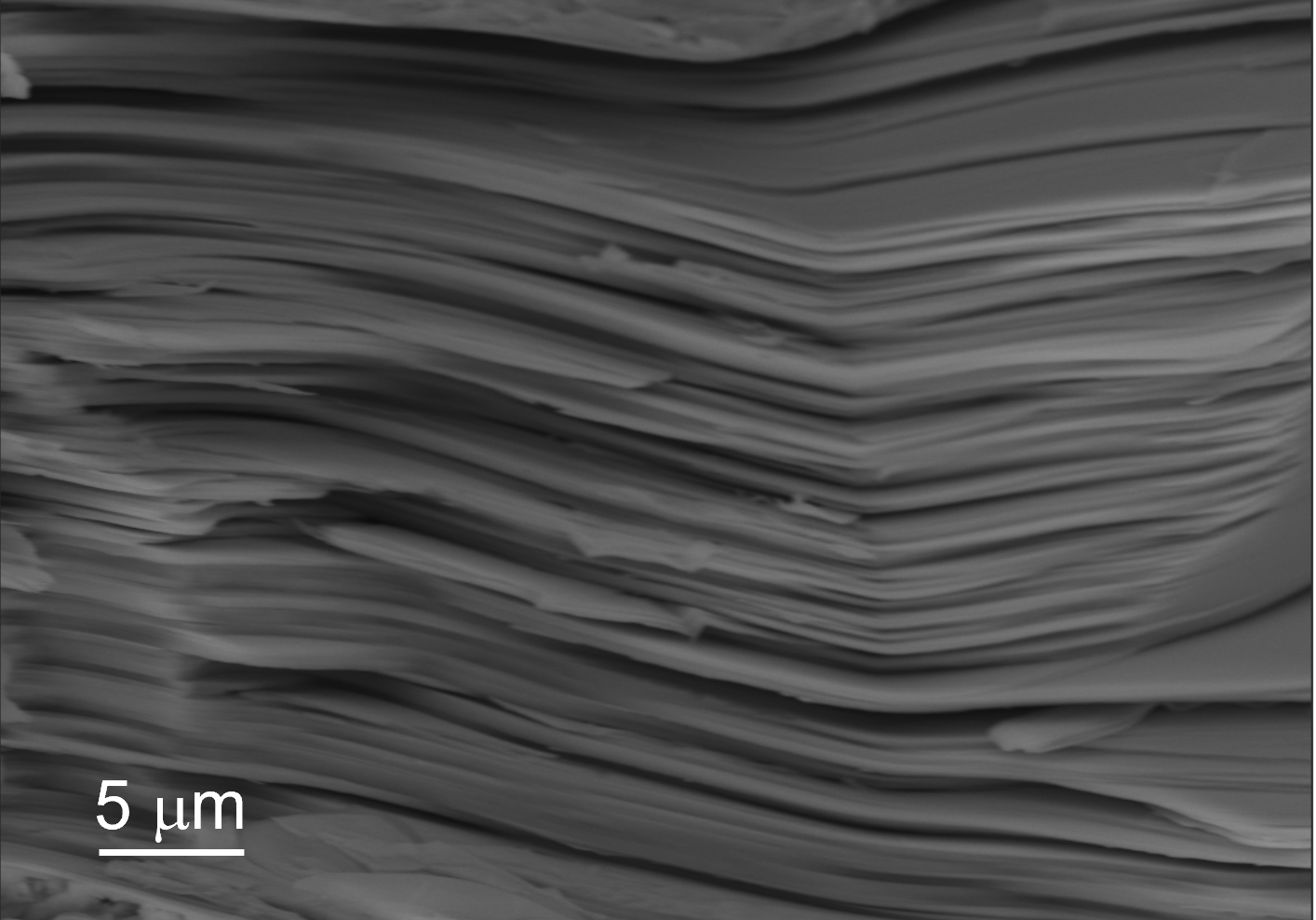


**Figure S2**. The scanning electron microscope (SEM) image of SnBi_2_Te_4_.


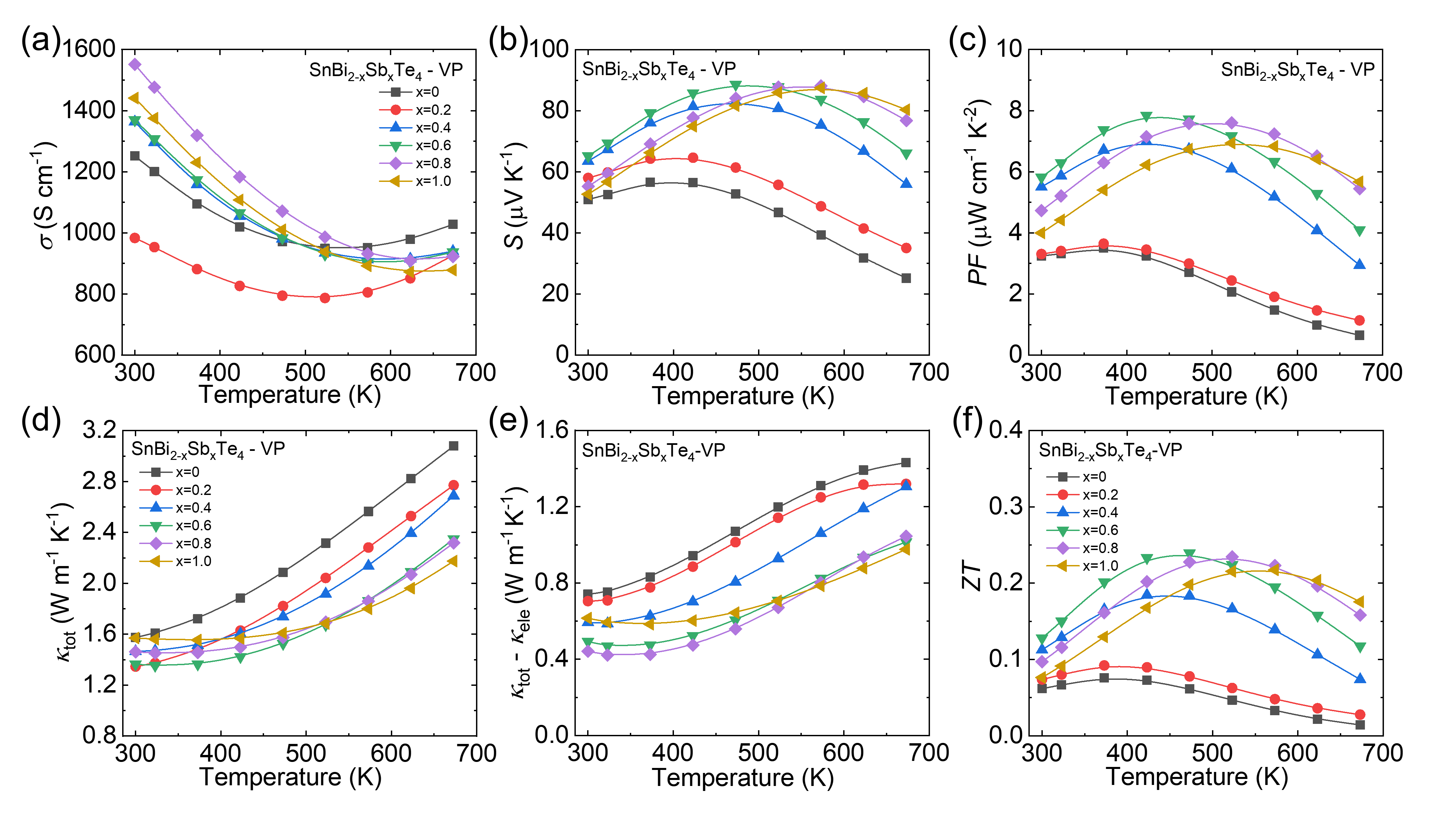


**Figure S3**. Electrical and thermal properties of SnBi_2-x_Sb_x_Te_4_(x = 0 - 1.0) along the perpendicular direction:(a) electrical conductivity, (b) Seebeck coefficient, (c) power factor, (d) total thermal conductivity, (e) summation of lattice and bipolar diffusion thermal conductivity, and (f) *ZT* value.


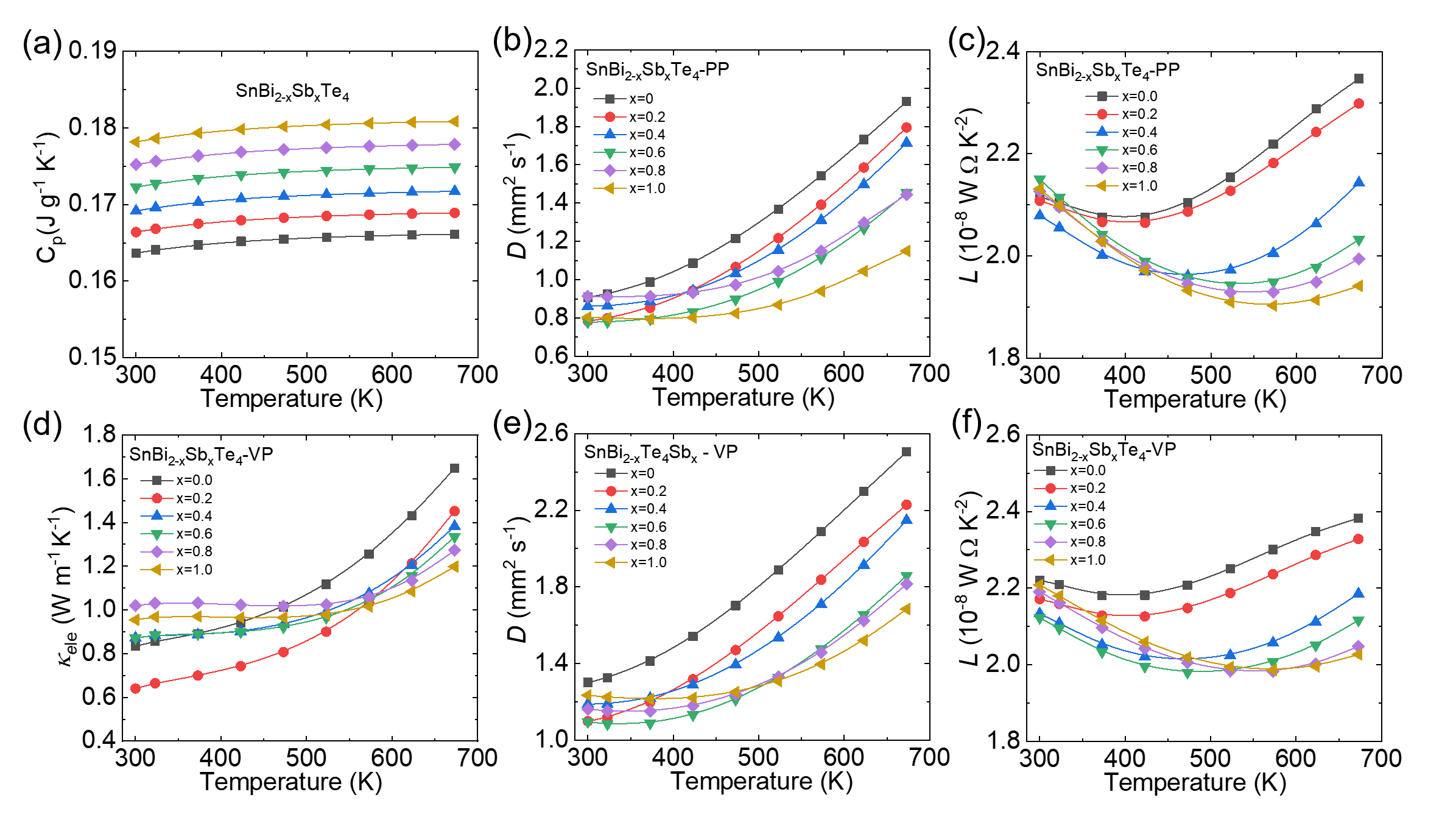


**Figure S4**. Thermal related properties of SnBi_2-x_Sb_x_Te_4_ (x = 0 - 1.0). (a) Heat capacity, (b) thermal diffusivity and (c) Lorentz number along the parallel direction. (d) Electronic thermal conductivity, (e) thermal diffusivity, (f) Lorentz number along the perpendicular direction.


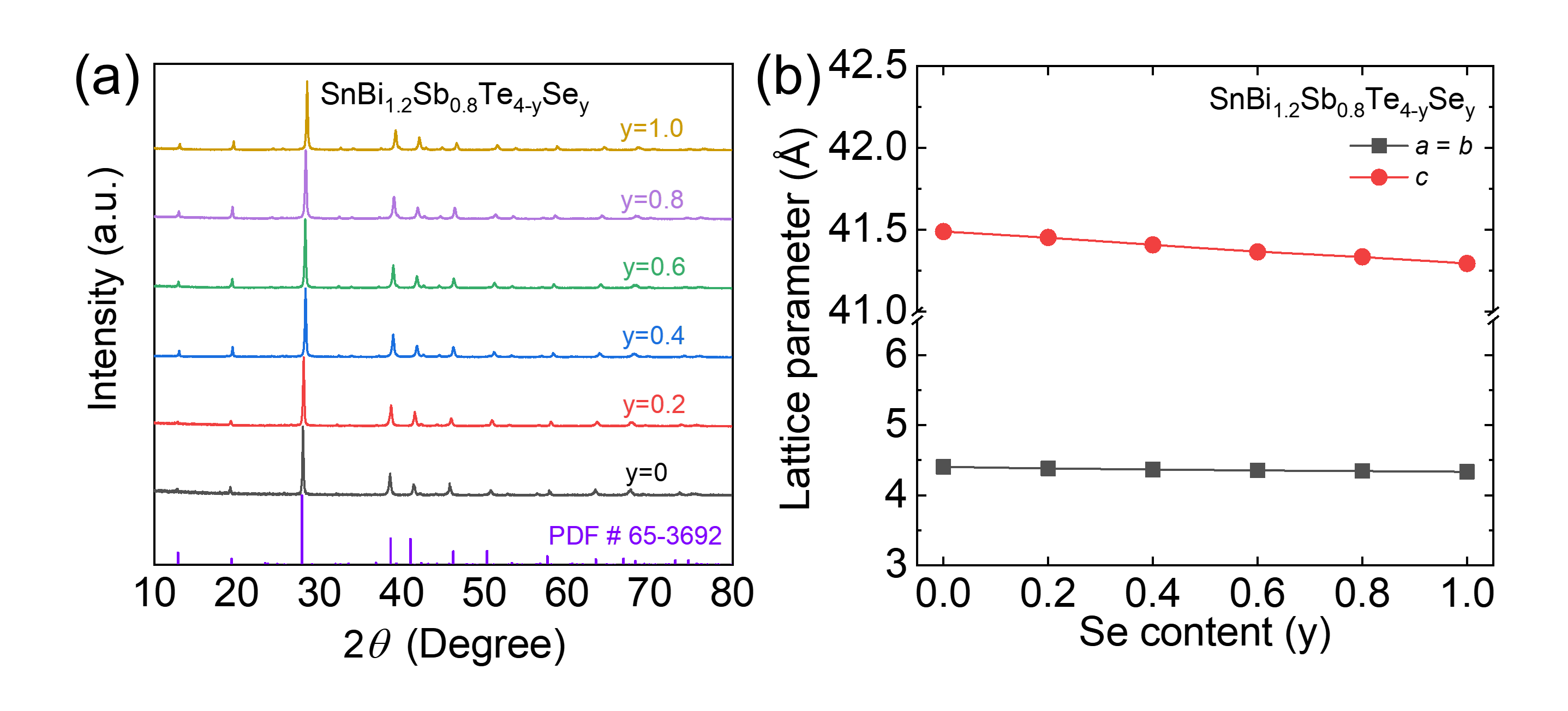


**Figure S5**. (a) The powder XRD and (b) the refined lattice parameters for SnBi_1.2_Sb_0.8_Te_4-y_Se_y_(y = 0 -1.0).


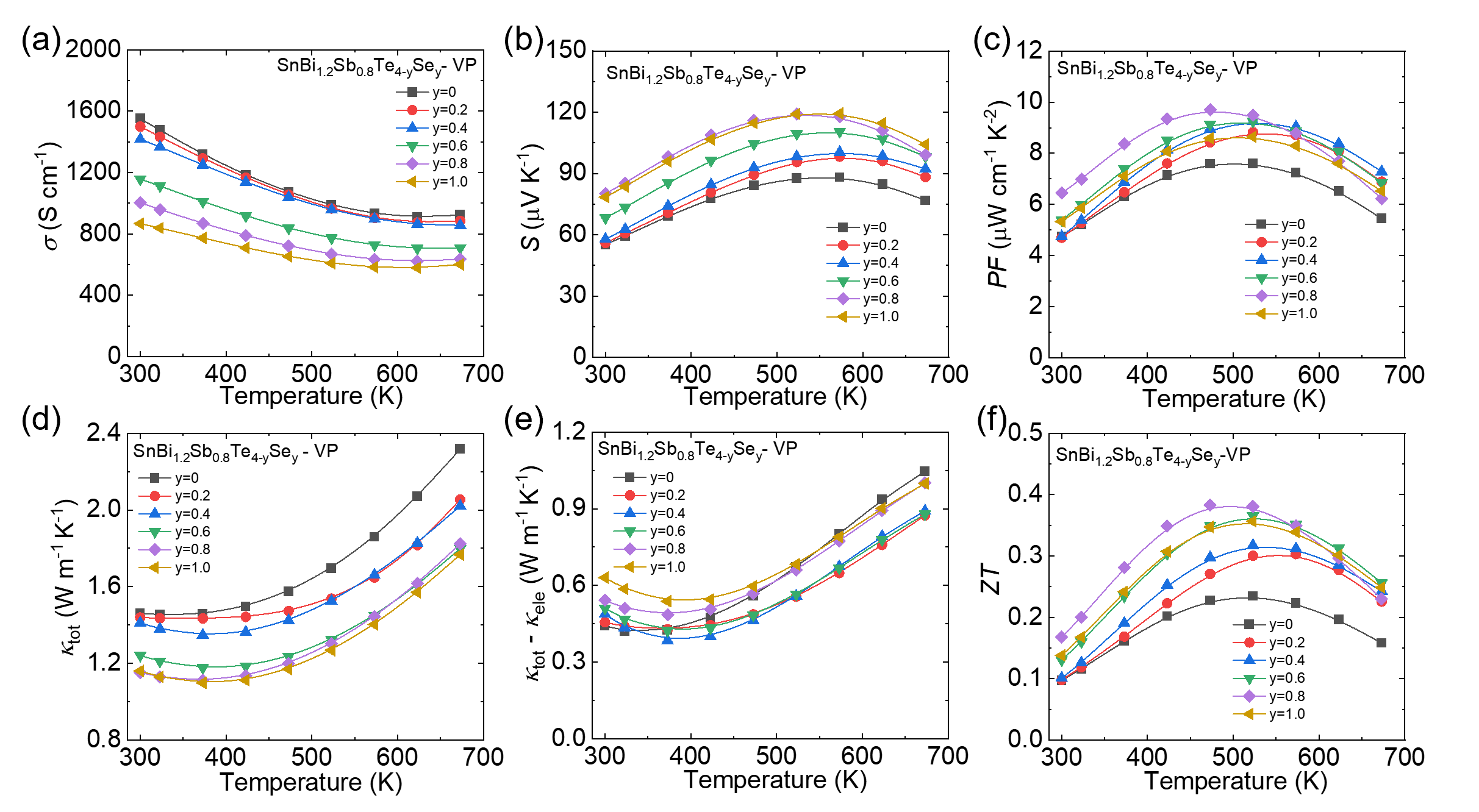


**Figure S6**. Thermoelectric transport properties of SnBi_1.2_Sb_0.8_Te_4-y_Se_y_ (y = 0 - 1.0) along the perpendicular direction: (a) electrical conductivity, (b) Seebeck coefficient, (c) power factor, (d) total thermal conductivity, (e) the summation of lattice and bipolar diffusion thermal conductivity, obtained by extracting the electronic part from total thermal conductivity *κ*_lat_ + *κ*_bi_ = *κ*_tot_ - *κ*_ele_ and (f) *ZT.*


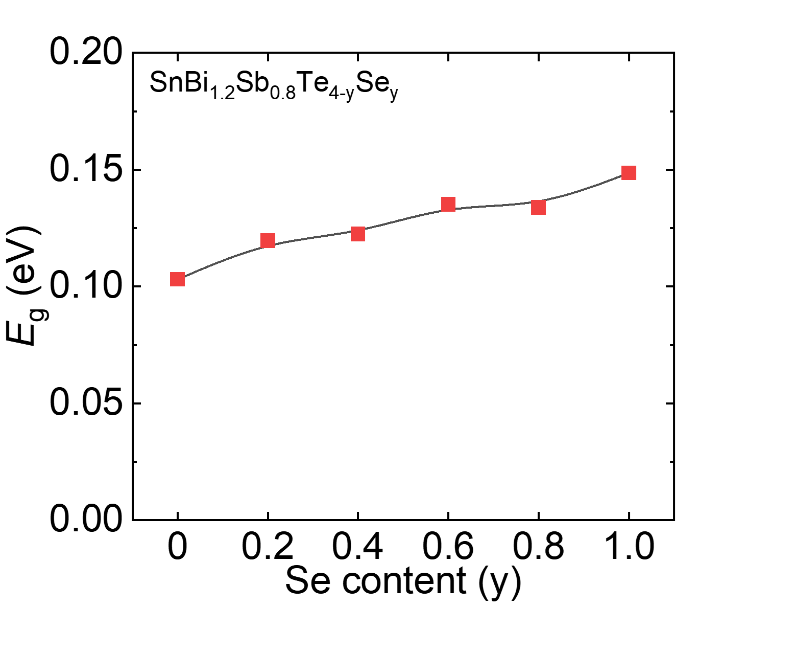


**Figure S7**. The band gaps evaluated from the relationship: *E*_g_ = 2*S*_max_*T*_max_.


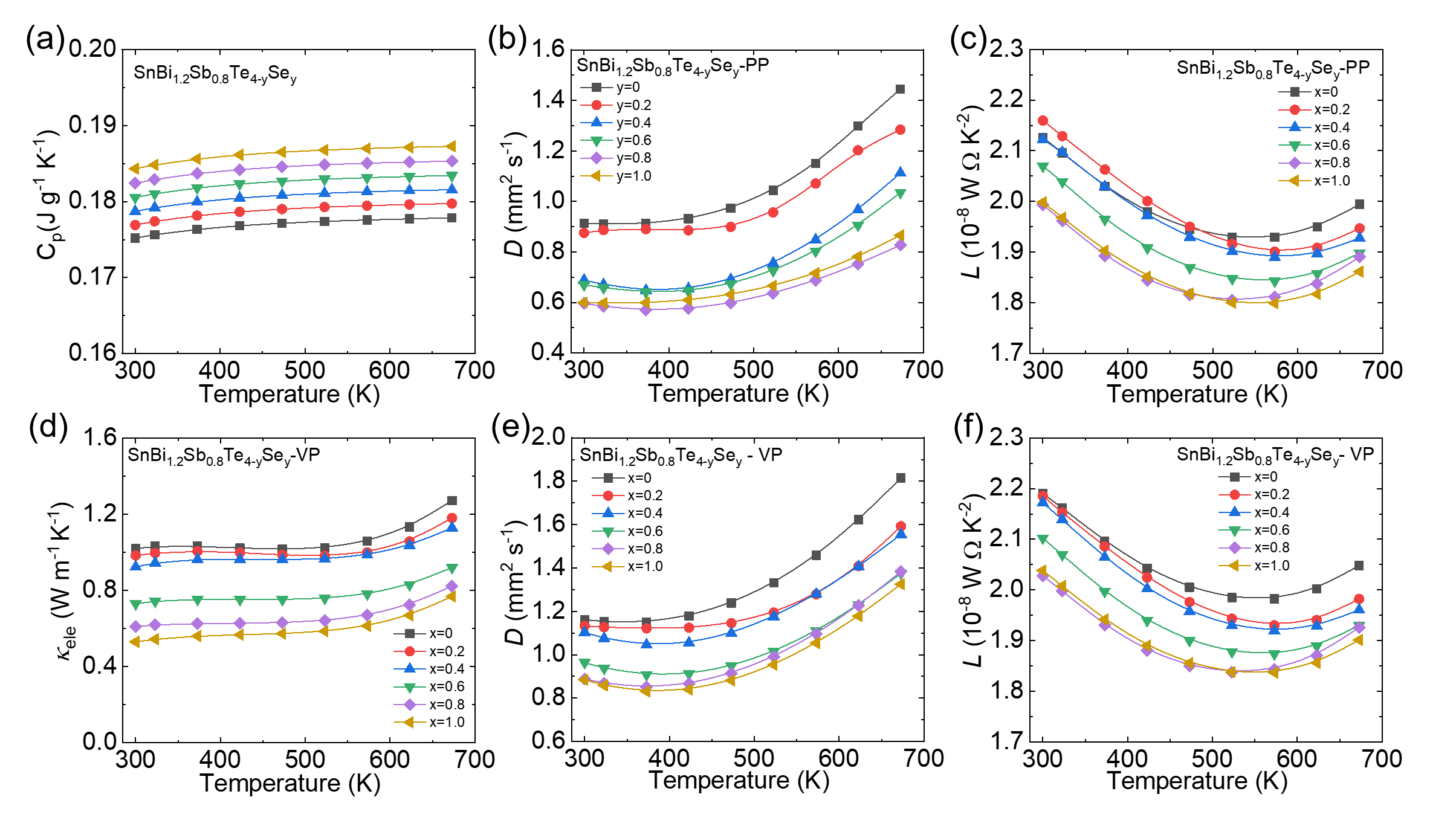


**Figure S8**. The thermal transport related properties of SnBi_1.2_Sb_0.8_Te_4-y_Se_y_ (y = 0 - 1.0): (a) Heat capacity. (b) Thermal diffusivity and (c) Lorentz number along the parallel direction. (d) Electronic thermal conductivity, (e) thermal diffusivity, and (d) Lorentz number along the perpendicular direction.

**Table S1**. Sample density of SnBi_2-x_Sb_x_Te_4-y_Se_y_ (x = 0 - 1.0; y = 0 - 1.0).

| Samples | ρ（g/cm^3^) |
| --- | --- |
| SnBi_2_Te_4_ | 7.40 |
| SnBi_1.8_Sb_0.2_Te_4_ | 7.36 |
| SnBi_1.6_Sb_0.4_Te_4_ | 7.29 |
| SnBi_1.4_Sb_0.6_Te_4_ | 7.23 |
| SnBi_1.2_Sb_0.8_Te_4_ | 7.18 |
| SnBi_1.2_Sb_0.8_Te_3.8_Se_0.2_ | 7.18 |
| SnBi_1.2_Sb_0.8_Te_3.6_Se_0.4_ | 7.16 |
| SnBi_1.2_Sb_0.8_Te_3.4_Se_0.6_ | 7.13 |
| SnBi_1.2_Sb_0.8_Te_3.2_Se_0.8_ | 7.11 |
| SnBi_1.2_Sb_0.8_Te_3_Se | 7.12 |

**References**

[1] X. Y. Liu; D. Y. Wang; H. J. Wu; J. F. Wang; Y. Zhang; G. T. Wang; S. J. Pennycook; L. D. Zhao, *Adv. Funct. Mater.* **2019**, *29*, 1806558.

[2] Y. Z. Pei; A. D. LaLonde; N. A. Heinz; X. Y. Shi; S. Iwanaga; H. Wang; L. D. Chen; G. J. Snyder, *Adv. Mater.* **2011**, *23*, 5674-+.

[3] J. Guo; Y. X. Zhang; Z. Y. Wang; F. S. Zheng; Z. H. Ge; J. C. Fu; J. Feng, *Nano Energy* **2020**, *78*, 105227.

[4] G. J. Snyder; A. H. Snyder; M. Wood; R. Gurunathan; B. H. Snyder; C. Niu, *Adv. Mater.* **2020**, *32*, 2001537.

[5] B. Jiang; X. Liu; Q. Wang; J. Cui; B. Jia; Y. Zhu; J. Feng; Y. Qiu; M. Gu; Z. Ge; J. He, *Energy Environ. Sci.* **2020**, *13*, 579-591.
